# The role of lysosomes and autophagosomes in frontotemporal lobar degeneration

**DOI:** 10.1111/nan.12500

**Published:** 2018-06-19

**Authors:** H. D. C. Bain, Y. S. Davidson, A. C. Robinson, S. Ryan, S. Rollinson, A. Richardson, M. Jones, J. S. Snowden, S. Pickering‐Brown, D. M. A. Mann

**Affiliations:** ^1^ Division of Neuroscience and Experimental Psychology School of Biological Sciences Faculty of Biology, Medicine and Health University of Manchester Salford Royal Hospital Salford UK; ^2^ Division of Neuroscience and Experimental Psychology School of Biological Sciences Faculty of Biology, Medicine and Health University of Manchester Manchester UK; ^3^ Cerebral Function Unit Greater Manchester Neurosciences Centre Salford Royal Hospital Salford UK

**Keywords:** autophagosomes, frontotemporal lobar degeneration, lysosomes

## Abstract

**Introduction:**

Cell biological and genetic evidence implicate failures in degrading aggregating proteins, such as tau and TDP‐43, through the autophagy or lysosomal pathways in the pathogenesis of frontotemporal lobar degeneration (FTLD).

**Methods:**

We investigated changes in the degradative pathways in 60 patients with different pathological or genetic forms of FTLD employing immunohistochemistry for marker proteins such as lysosomal‐associated membrane proteins 1 (LAMP‐1) and 2 (LAMP‐2), cathepsin D (CTSD) and microtubule‐associated protein 1 light chain 3 alpha (LC3A). Immunostained sections were qualitatively and semi‐quantitatively assessed for the appearance, distribution and intensity of staining in neurones of the dentate gyrus (DG) and CA4 region of the hippocampus, and the temporal cortex (Tcx).

**Results:**

Lower levels of neuronal LAMP‐1 immunostaining were present in the DG and Tcx in FTLD‐tau compared to FTLD‐TDP. There was less LAMP‐1 immunostaining in FTLD‐tau with *MAPT* mutations, and FTLD‐tau with Pick bodies, compared to FTLD‐TDP types A and B, and less LAMP‐1 immunostaining in FTLD‐TDP type C than in FTLD‐TDP types A and B. There was greater LAMP‐1 immunostaining in *GRN* mutation which may reflect the underlying type A histology rather than mutation. There were no differences in neuronal LAMP‐2, CTSD, EEA‐1 or LC3A immunostaining between any of the five FTLD histological or four genetic groups, nor between FTLD‐TDP and FTLD‐tau.

**Conclusions:**

The underlying pathological mechanism in FTLD‐tau may lie with a relative deficiency of lysosomes, or defective vesicular transport, whereas the failure to clear TDP‐43 aggregates may lie with lysosomal dysfunction rather than a lack of available lysosomes or degradative enzymes.

## Introduction

Frontotemporal lobar degeneration (FTLD) describes a spectrum of neurodegenerative disorders with heterogeneous clinical presentation, pathology and genetic abnormalities, which result in atrophy of the frontal and temporal lobes. The major clinical syndromes involve personality and behavioural changes (behavioural variant frontotemporal dementia, or bvFTD) or language alterations of a fluent (semantic dementia) or nonfluent (progressive nonfluent aphasia) nature 1. All three syndromes can be accompanied by amyotrophic lateral sclerosis (ALS), although the combination of bvFTD+ALS is most common 1, 2. Although the disease mechanisms underlying FTLD remain unknown, genetic associations may provide clues to pathogenesis. Major causative mutations have been identified in tau (*MAPT*) 3, progranulin (*GRN*) 4, 5 and more recently associated with hexanucleotide expansion in *C9orf72* 6, 7. However, rarer forms of FTLD and/or ALS have been associated with mutations in valosin‐containing protein (*VCP*) 8, *CHMP2B* 9, ubiquilin 1(*UBQLN1*) 10, optineurin (*OPTN*) 11 and sequestosome 1 (*SQSTM1*) (also known as p62) 12 genes. Genome‐wide association studies have implicated variations in *TMEM106B* as a risk factor for FTLD 13.

Pathologically, about 45% of cases of FTLD show tauopathy, involving neurofibrillary tangle‐like structures or Pick bodies, whereas approximately 50% of cases show inclusion bodies within neurones (NCI) and/or dystrophic neurites (DN) in the cerebral cortex and in the hippocampus that contain the nuclear transcription factor, TDP‐43 14; around 5% of cases display NCI composed of fused in sarcoma protein. Accumulation of these structurally modified proteins is considered to reflect a failure on the part of neuronal clearance mechanisms which internally degrade such proteins and/or eliminate them from the cell. Neurones employ several protein clearance strategies which include:

- Endoplasmic‐reticulum‐associated degradation (ERAD) in which misfolded proteins are recognized in the endoplasmic reticulum and tagged with ubiquitin 15.
- The ubiquitin–proteasome system (UPS) in which the polyubiquitin‐tagged proteins are chaperoned to the proteasome and enzymatically cleaved into amino acids 16.
- Macroautophagy in which protein aggregates too large for UPS and ERAD are surrounded by vesicles (autophagosomes) and transported to lysosomes resulting in fusion and lysosomal enzymatic degradation 17, 18.
- Microautophagy in which aggregates/organelles are sequestered directly by the lysosome through invaginations from its membrane 19.
- Chaperone‐mediated autophagy (CMA) in which proteins are transported to the lysosome via chaperone proteins, such as Hsc70, in the absence of vesicular containment 20.
- Endosomal sorting in which endocytosed products are targeted back to the cell surface, to lysosomes for degradation or exported from the cell as exosomes 21.

There is good evidence that most, if not all, the genetic mutations that cause or increase the risk of FTLD encode proteins that participate in neuronal protein clearance pathways. For example, CHMP2B participates in the endosomal sorting complex regulating the fusion of the endosome with the autophagosome, and ultimately with the lysosome 9. Valosin‐containing protein is a multi‐ubiquitin chain‐targeting factor required in ubiquitin–proteasome degradation 22. Optineurin is a polyubiquitin‐binding adaptor protein which can interact with p62 and TANK1‐binding kinase protein in the regulation of autophagy 23. Sequestosome 1 (p62) is a key player in UPS 24. Ubiquilin‐1 functions in autophagy and is degraded by CMA 25. Progranulin (PGRN) is localized to lysosomes through at least two independent trafficking pathways 26, 27. Although the role of C9orf72 protein is unclear, it is believed to be implicated in membrane trafficking and autophagy 28, 29, 30. Furthermore, while heterozygous loss of function mutations in *GRN* cause FTLD‐TDP 4, 5, homozygous mutations in *GRN* cause a lysosomal storage disorder known as neuronal ceroid lipofuscinosis (NCL) 31, 32. Indeed, recent experimental work 33 indicates that mice homozygous for *GRN* mutation not only show evidence of a lysosomal disorder, similar to that seen in NCL, with elevated marker proteins, but also demonstrate TDP‐43 proteinopathy. Moreover, such mice also show increased levels of TMEM106B. Conversely, homozygous mutations in cathepsin D gene (*CTSD*) (a model of NCL) in mice result in increases in PGRN and TMEM106B proteins with pathological deposition of TDP‐43 33. These observations suggest that changes in lysosomal function might underpin the pathogenetic mechanism of both FTLD and NCL, and that PGRN and CTSD might function together in the regulation of lysosomal activity; a view supported by recent studies 34.

Hence, there is strong genetic and cell biological support for the view that disturbances in lysosomal and autophagosomal function, leading to accumulations of unwanted, potentially neurotoxic proteins, may underpin the pathological changes which characterize FTLD‐TDP, and particularly so in those cases bearing mutations in *GRN*. With this in mind, we have investigated lysosomal and autophagosomal function in 60 cases of FTLD, and in others with Alzheimer's disease (AD) or without significant pathology, these acting as neurodegenerative and healthy controls respectively. Sections of the temporal cortex with the hippocampus were immunostained for marker proteins such as lysosomal‐associated membrane proteins 1 and 2 (LAMP‐1 and LAMP‐2), cathepsin D (CTSD), EEA‐1 and the microtubule‐associated protein 1A/1B‐light chain α (LC3A). LAMP‐1 is a structural glycoprotein associated with the lysosomal membrane, but also shows colocalization with the autophagosomal marker LC3A, implying it has a role in autophagolysosome fusion 35. LAMP‐2 is essential for CMA. In this process, the chaperone protein Hsc70 binds to KFERQ motifs that are exposed when proteins suffer insults to their structural integrity 36. Hsc70 forms a translocation complex with LAMP‐2A creating a pore through which to thread the protein intended for degradation. CTSD is a lysosomal aspartic‐type protease which degrades aggregates delivered to the lysosome via CMA or endocytosis 37. EEA1 is a Rab5 effector protein that has a role in early endosome docking/fusion 38. Neurones of the temporal cortex (Tcx), dentate gyrus (DG) and CA4 regions of the hippocampus were therefore assessed qualitatively and semi‐quantitatively for the presence, intracellular distribution and relative staining intensity of these proteins according to pathological and genetic subtype. In so doing, protein degradation was assessed at different levels with LAMP‐1 acting as a marker of lysosomal number, LAMP‐2 as a marker of CMA and lysosomal function, CTSD as a marker of lysosomal protein degradation activity and LC3A as a marker of autophagosomal activity.

## Materials and methods

### Patients

The study included 72 subjects in total, comprising one group of 60 patients with a clinical diagnosis of FTLD (31 males, 24 females; cases #1–60), 6 with a diagnosis of AD (4 males, 2 females; cases #61–66) and 6 control subjects with no evidence of neurodegenerative disease (3 males, 7 females; cases #67–72) (see Table S1). The brains of these patients had been consecutively acquired by Manchester Brain Bank (MBB) over the years 1986 to present. All patients were from the North West of England and North Wales, and tissues were obtained through appropriate consenting procedures for the collection and use of the human brain tissues. All 60 FTLD patients fulfilled relevant clinical diagnostic criteria 39, 40, 41 having been investigated longitudinally within specialist dementia clinics at Salford Royal Hospital using the Manchester Neuropsychological Profile (Man‐NP) 42, 43 to determine and characterize the nature of their dementia.

Of the 60 FTLD patients, 32 had been clinically diagnosed with bvFTD (20 males, 32 females), 9 with bvFTD+ALS (6 males, 3 females), 11 with progressive nonfluent aphasia (PNFA) (9 males, 2 females), 7 with semantic dementia (SD) (3 males, 4 females) and 1 with progressive supranuclear palsy (PSP) and ALS (see Table S1). Histologically, the FTLD group comprised of 24 patients with FTLD‐TDP type A (cases #1–7, 14–30), 17 with FTLD‐TDP type B (cases #8–13, 31–41), 7 with FTLD‐TDP type C (cases #42–48) and 12 with FTLD‐tau (cases #49–60) of which 6 were associated with exon 10 +16 splice mutation (cases #49–54) and 6 with Pick's disease histology (cases #55–60) 44. Furthermore, within the FTLD‐TDP group, there were 13 patients with expansions in *C9orf72* (cases #1–13), 12 with *GRN* mutations (cases #14–25) and 23 without known mutation (cases #25–48).

Comparison of the five FTLD pathology patient groups showed significant differences in the mean age at onset of disease (*F*_4,55_ = 3.2, *P* = 0.02), mean age at death (*F*_4,55_ = 4.8, *P* = 0.002) but duration of illness did not differ (*F*_4,55_ = 1.7, *P* = 0.156). Patients with FTLD‐tau with *MAPT* mutation had earlier age at onset than those with the FTLD‐TDP type A and FTLD‐TDP type C subgroups (*P* = 0.014 and 0.044, respectively), but not with the FTLD‐TDP type B subgroup (*P* = 0.849) or Picks group (*P* = 0.646). Patients with FTLD‐tau also had earlier age at death than those with the FTLD‐TDP type A and FTLD‐TDP type C subgroups (*P* = 0.007 in both instances), but not with the FTLD‐TDP type B subgroup (*P* = 0.143) or Picks group (*P* = 0.369) (Table 1).

**Table 1:** Selected clinical, neuropathological and genetic details on patients studied

Pathological group	M/F	Age at onset (y)	Age at death (y)	Duration of illness (y)
FTLD‐TDP type A (*n* = 24)	16/8	61.6 ± 6.5	70.0 ± 8.3	8.3 ± 3.8
FTLD‐TDP type B (*n* = 17)	13/4	58.9 ± 7.9	64.8 ± 8.8	5.9 ± 4.6
FTLD‐TDP type C (*n* = 7)	3/4	62.3 ± 7.1	72.4 ± 5.9	10.1 ± 4.6
FTLD‐TDP *C9orf72* expansion (*n* = 13)	9/4	60.8 ± 7.6	67.1 ± 6.8	6.1 ± 4.7
FTLD‐TDP *GRN* mutation (*n* = 12)	7/5	59.9 ± 5.1	69.4 ± 3.4	9.5 ± 4.2
FTLD‐TDP No mutation (*n* = 23)	16/7	60.3 ± 8.6	67.9 ± 9.0	7.9 ± 3.9
FTLD‐tau *MAPT* mutation (*n* = 6)	2/4	50.5 ± 4.2	58.7 ± 4.6	8.2 ± 3.4
FTLD‐tau Pick's disease	5/1	57.0 ± 11.2	66.0 ± 8.9	9.0 ± 3.3
Alzheimer's disease (*n* = 6)	4/2	64.2 ± 5.9	74.3 ± 3.7	9.8 ± 3.0
Controls (*n* = 6)	3/3	na	79.3 ± 14.9	na

FTLD, frontotemporal lobar degeneration.

Comparison of the four FTLD genetic patient groups also showed significant differences in the mean age at onset of disease (*F*_3,56_ = 3.1, *P* = 0.032) and mean age at death (*F*_3,56_ = 3.2, *P* = 0.030), though duration of illness did not differ significantly (*F*_3,56_ = 1.5, *P* = 0.236). Patients with *MAPT* mutation had earlier age at onset than those with *C9orf72* expansion (*P* = 0.035) and those without known mutation (*P* = 0.025), but not to those with *GRN* mutation (*P* = 0.069) which in turn did not differ from *C9orf72* (*P* = 0.990) and no mutation groups. The mean age at death was significantly earlier in the *MAPT* than in the *GRN* (*P* = 0.026) and no mutation (*P* = 0.999) groups (*P* = 0.028) which in turn did not differ from the *C9orf72* (*P* = 0.858) and no mutation (*P* = 0.964) groups (Table 1).

### Histological methods

Paraffin sections were cut at 6 μm from formalin fixed blocks of the temporal lobe (BA21/22) (to include the posterior hippocampus) from all 60 FTLD cases, 6 AD cases and the 6 control cases. Following titration (dilutions 1:100 to 1:2000) to determine optimal immunostaining, antibodies were identically employed in a standard IHC protocol, as described previously 45, 46. The following antibodies were employed: rabbit polyclonal LAMP‐1 antibody (Abgent, San Diego, USA; AP1823a, 1:750), mouse monoclonal LAMP‐2 antibody (Aviva Systems Biology, San Diego, USA; OAAB06711, 1:4000), rabbit polyclonal CTSD antibody (Insight Biotechnology Ltd, Wembley, UK; sc‐10725, 1:200), rabbit polyclonal EEA‐1 (Abcam, Cambridge UK; ab2900, 1:2000) and rabbit polyclonal anti‐microtubule‐associated protein 1 light chain 3 alpha (LC3A) antibody (Sigma‐Aldrich, St Louis, USA; L8793, 1:2000). For each antibody, antigen unmasking was performed by pressure cooking in citrate buffer (pH 6.0, 10 mM) over a 30‐min period to include warming and cooling times, reaching 123°C for 30 s, and >15 psi pressure.

In order to investigate the possible colocalization of TDP‐43 protein within the autophagosomal/lysosomal pathway (ALP), immunoreactive structures double immunofluorescence staining was performed using a human‐specific monoclonal TDP‐43 antibody (Proteintech, Manchester, UK, 60019‐2‐Ig, at 1:2000) and the aforementioned polyclonal LAMP‐1 antibody (Abgent, San Diego, USA; AP1823a, at 1:300 dilution) as representative of the ALP markers. Briefly, tissue sections were deparaffinated in xylene and dehydrated in ethanol. Antigen retrieval was achieved by boiling for 5 min in 10 mM trisodium citrate buffer with 0.05% Tween‐20 v/v (pH6.5) using a pressure cooker. Sections were cooled slowly and washed three times in phosphate‐buffered saline (PBS) before blocking in 1% fish skin gelatine in PBS for 1 h at room temperature. Three more PBS washes were performed. Slides were immunolabelled using rabbit polyclonal anti‐LAMP1 antibody and mouse anti‐TDP‐43 antibody. Primary antibody incubations were performed overnight at 4°C and removed by washing with PBS three times. Secondary antibody incubations were performed for 1 h at room temperature using donkey anti‐mouse IgG conjugated to AlexaFluor‐488 and donkey anti‐Rabbit IgG conjugated to AlexaFluor‐594 (Life Technologies; 1 drop in 500 μl PBS). Sections were washed in PBS, treated with 1% Sudan Black B w/v in ethanol for 5 min and washed an additional three times in PBS with a final wash in water before drying overnight. 4′‐6‐Diamidino‐2‐phenylindol (DAPI) was used for nuclear counterstaining. Coverslips were mounted using a ProLong Diamond Antifade mountant with DAPI (Thermo Fisher). Images were ascertained under oil immersion on a Zeiss Axioimager D2 upright microscope using a 63x*/*1.4 EC Plan‐ApoChromat objective and captured using a Coolsnap HQ2 camera (Photometrics) through Micromanager software v1.4.23. Specific band pass filter sets for DAPI, FITC and Texas red were used to prevent bleed through from one channel to the next. Images were processed and analysed using Fiji ImageJ (http://imagej.net/Fiji/Downloads).

### Pathological assessment

Sections were examined microscopically for the appearance of intracellular distribution of immunostaining within neurones of the Tcx, DG and CA4 region of the hippocampus and for the presence of any immunostained structures (NCI) resembling those seen in TDP‐43 or tau‐immunostaining. These regions were chosen because the Tcx and DG of the hippocampus are involved with TDP‐43 pathology in all forms of FTLD‐TDP, or tau pathology in those patients with *MAPT* mutation. Moreover, the CA4 region of the hippocampus was included along with the DG as these are among the principal regions affected by DPR pathology in patients with expansions in *C9orf72* 45, 46.

Following immunostaining, images of the DG, CA4 and inferior temporal gyrus were captured at x40 microscope objective magnification, using a Leica DMR light microscope, and subsequently analysed on Corel PaintShop Pro X7 which allowed significant zoom in order to assist in distinguishing between similar levels of staining intensity.

A four‐point grading system was employed (see Figure S1):

- Grade 0 – no cells stained or any staining that could be considered to be above background level (i.e. 1–2 cells very lightly stained).
- Grade 1 – some cells (2+) very lightly stained with a maximum of one moderately stained.
- Grade 2 – most cells moderately stained with one or two heavily staining.
- Grade 3 – most or all cells heavily stained.

In order to minimize intra‐observer subjectivity and maximize accuracy of grading semi‐quantitative analyses for each pathological measure were performed by a single investigator (HB or DMAM) who also devised the grading systems employed. For each assessment, cases were graded three times blind to previous results and any discrepancies recorded. Discrepancies were reviewed closely, and scores and grading systems were adjusted by consensus. Random samples for each assessment were subsequently taken and re‐graded blind to previous results obtained to test the reproducibility of the scores and to test the reliability of the grading systems. Moreover, to assess inter‐observer variability, a subset of 20 immunostained cases for each measure, selected at random, was chosen for independent scoring by both observers. This showed good agreement between all pairs of scores (κ = 0.65, *P* = 0.000), with 75% of scores being the same, and no score between cases differing by more than one grade in any pairwise comparison. Hence, use of these particular scoring systems show robust agreement when employed either by both highly (DMAM) or lesser experienced (HB) observers.

### Statistical analysis

Rating data was entered into an excel spreadsheet and analysed using Statistical Package for Social Sciences (SPSS) software (version 17.0). The FTLD patients were stratified according to genetic and pathological subtype for statistical analysis of the effect of each mutation and underlying pathology on the pattern of the staining for each antibody. Comparisons of semi‐quantitative scores for intensity of immunostaining in the Tcx, DG and CA4 region of the hippocampus were all performed using the Kruskal–Wallis test with the post hoc Mann–Whitney test where Kruskal–Wallis yielded a significant difference between antibody staining scores. Group comparisons of age at onset, age at death and duration of illness were made using an ANOVA with a post hoc Tukey test. In all instances, significance levels were set at *P* < 0.05. Correlation analysis (Spearman rank correlation test) was performed between scores for all five antibodies in each of the three regions investigated. Correlations were performed for all FTLD cases combined, and when stratified into all FTLD‐TDP or FTLD‐tau cases. In order to avoid type 1 errors from multiple testing, significance levels were stringently set at *P* < 0.01.

## Results

### Cytological appearance

**Figure 1:**
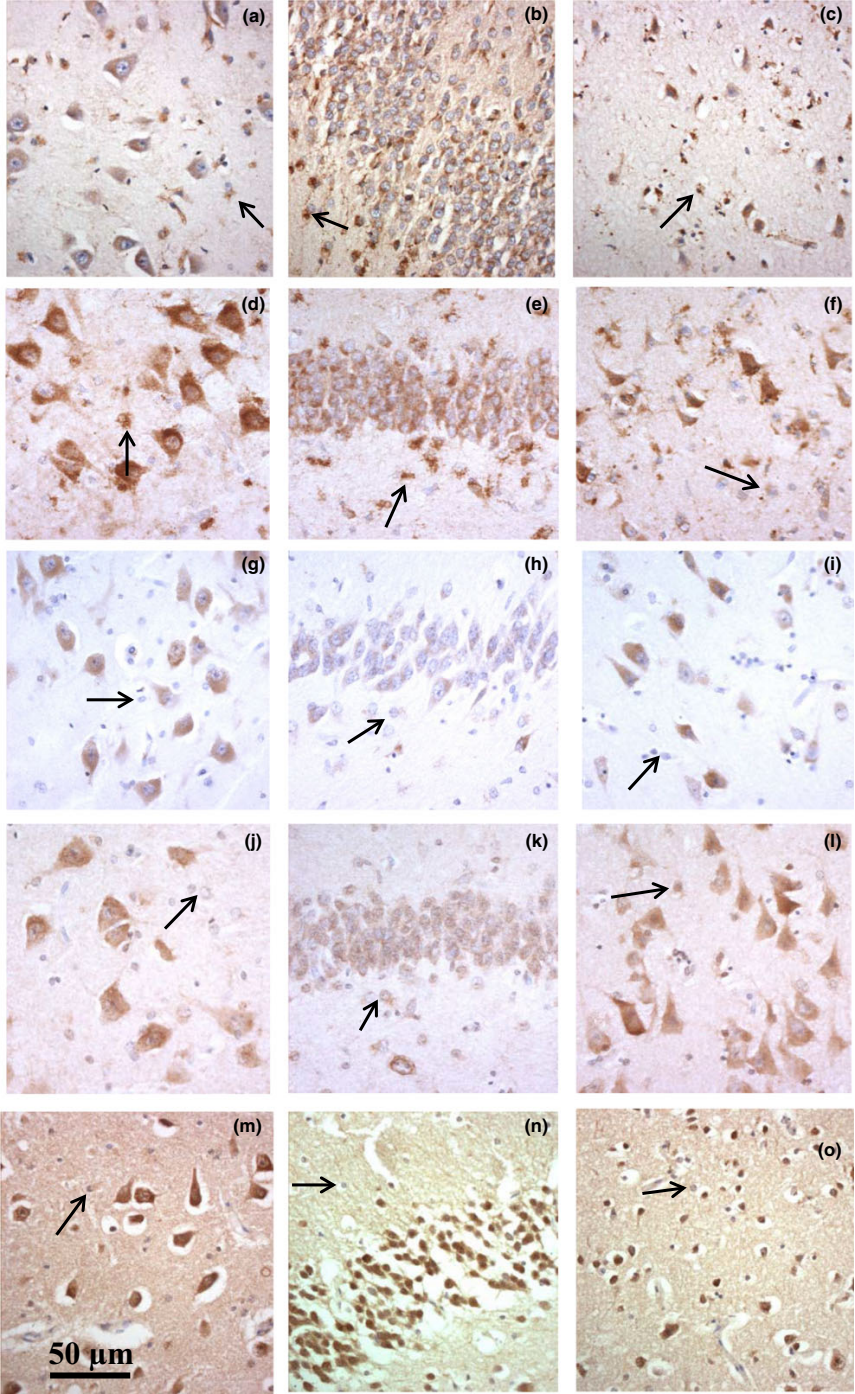
Immunostaining for LAMP‐1 (**a**–**c**), LAMP‐2 (**d**–**f**), CTSD (**g**–**i**), EEA‐1 (**j**–**l**) and LC3A (**m**–**o**) in neurones and glia (arrowed) in the CA4 (**a, d, g, j** and **m**) and dentate gyrus (**b, e, h, k** and **n**) regions of the hippocampus and temporal cortex (**c, f, i, l** and **o**) in controls (case #69). Immunostaining is present only in cell cytoplasm and is diffuse and granular in nature.

In control individuals, immunostaining for LAMP‐1, LAMP‐2, CTSD and EEA‐1 was present within the cell cytoplasm of neurones and glial cells, and in every instance appeared diffuse and granular in appearance (Figure 1). Nonetheless, immunostaining varied widely in intensity between the different ALP markers. For LAMP‐1 (Figure 1 **a**–**c**), LAMP‐2 (Figure 1 **d**–**f**) and LC3A (Figure 1 **m**–**o**), this was mostly moderate to strong, and present throughout the cell cytoplasm with some cases displaying a more intense perinuclear staining. However, CTSD (Figure 1 **g**–**i**) and EEA‐1 (Figure 1 **j**–**l**) immunostaining was less strong with most cases being only mild to moderately immunostained. LC3A immunostaining was more diffusely spread throughout the cell cytoplasm, sometimes with increased perinuclear staining intensity and significant ‘background’ staining (Figure 1 **m**–**o**) and was more uniform in intensity between cases.

**Figure 2:**
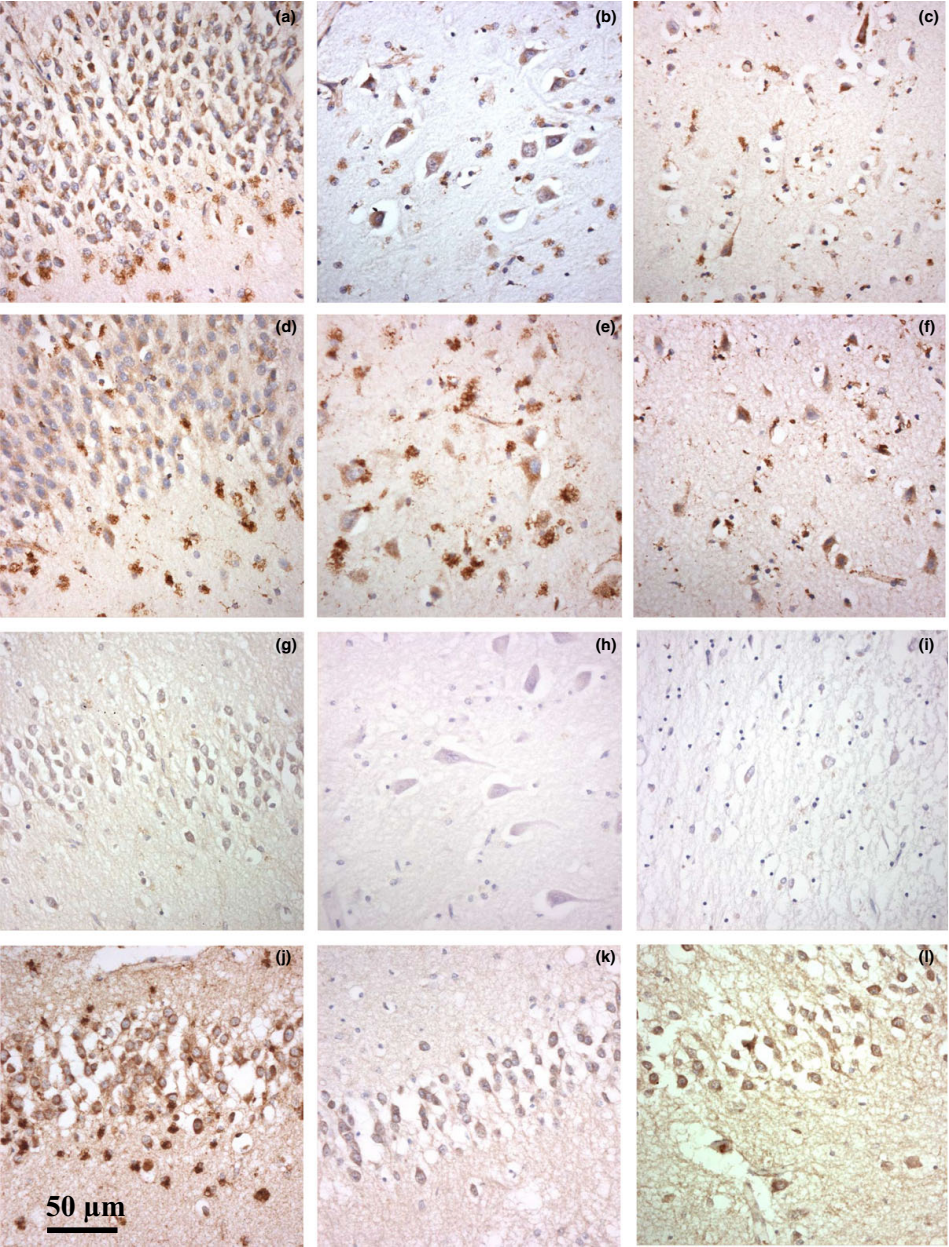
Immunostaining of neurones of the dentate gyrus (**a, d, g, j**–**l**), CA4 (**b, e, h**) and temporal cortex (**c, f, i**) for LAMP‐1 in FTLD‐TDP type A (case #7) (**a**–**c**), type B (case #12) (**d**–**f**) and type C (case #42) (**g**–**l**), and neurones of the dentate gyrus in FTLD‐TDP type C (case#42) for LAMP‐2 (**j**), CTSD (**k**) and LC3A (**l**). There is loss of LAMP‐1 immunostaining from all the three regions in FTLD‐TDP type C cases compared to types A and B with preservation of immunostaining for LAMP‐2, CTSD and LC3A.

**Figure 3:**
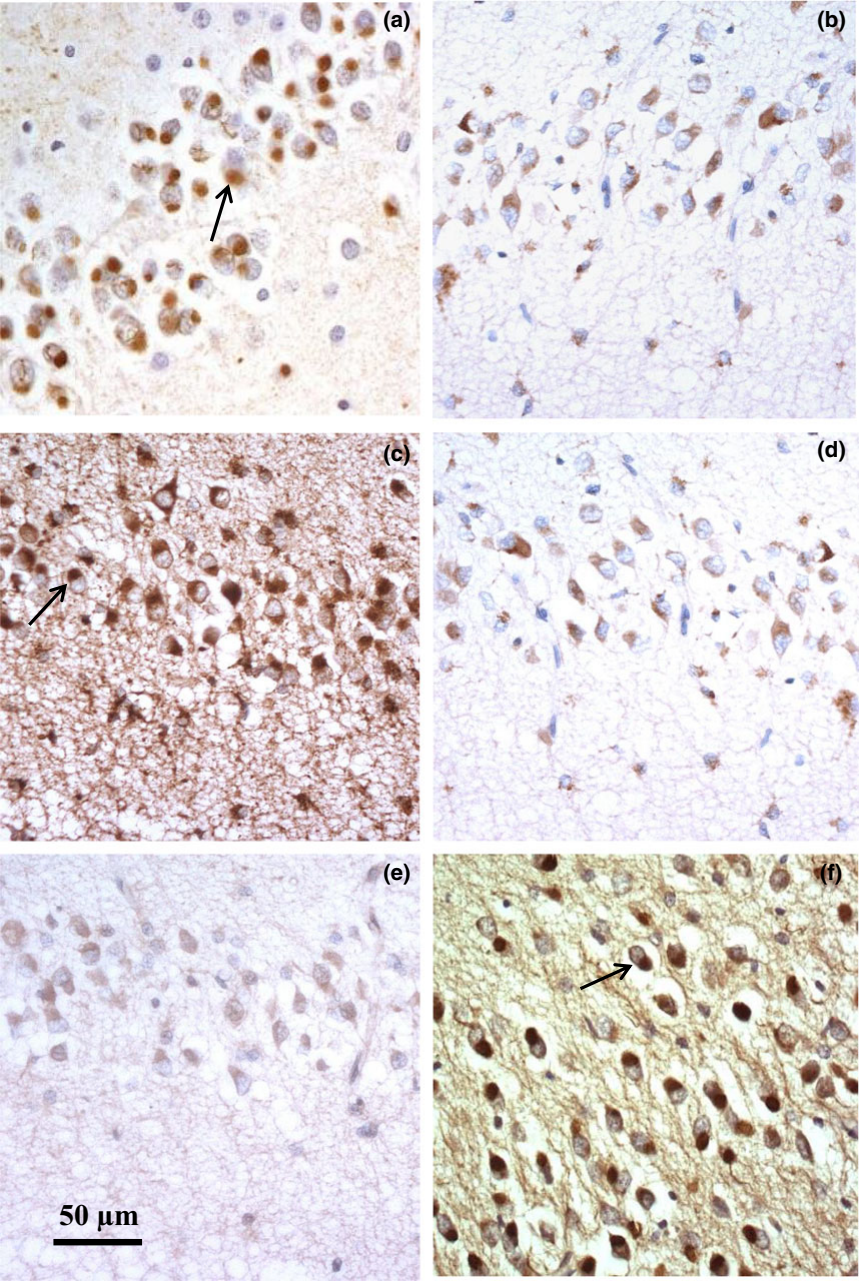
Immunostaining of Pick bodies for tau (**a**), LAMP‐1 (**b**), LAMP‐2 (**c**), CTSD (**d**), EEA‐1 (**e**) and LC3A (**f**) in neurones of the dentate gyrus of the hippocampus in FTLD‐tau (case #56). Tau‐positive Pick bodies (arrowed in (**a**)) are robustly immunostained for LC3A (arrowed in (**f**)), less clearly immunostained for LAMP‐2 (arrowed in (**c**)), but not at all for LAMP‐1 (**b**), CTSD (**d**) or EEA‐1 (**e**) where the immunostaining present is that of background lysosomal structures.

In general, there were no obvious microscopic differences from controls in the intensity or cellular distribution of immunostaining with any of the five ALP markers in most FTLD‐TDP or FTLD‐tau cases (Figures 2 and 3, respectively). The main exceptions were in respect of FTLD‐TDP cases with type C histology where there was noticeably less immunostaining for LAMP‐1 in the CA4, DG and Tcx (Figure 2 **g**–**i**), compared to that present in FTLD‐TDP type A (Figure 2 **a**–**c**) and type B (Figure 2 **d**–**f**) cases. Nonetheless, in these type C cases immunostaining for LAMP‐2, CTSD, EEA‐1 and LC3A in the same cell types was well maintained (Figure 2 **j**–**l**).

Although neuronal cytoplasm was (variably) immunostained for each of the ALP markers in all FTLD‐TDP cases (Figure 2), no immunostaining of any structures within neurones of the DG or Tcx unequivocally resembling TDP‐43 inclusion bodies was seen. This observation was borne out by double immunofluorescence which showed that markers for ALP did not contain TDP‐43 protein (see Figure S2).

Similarly, although the cytoplasm of neurones containing Pick bodies in FTLD‐tau cases (Figure 3 **a**) was also (often strongly) immunostained by each of the ALP markers (Figure 3 **b**–**f**), rounded structures, bearing a close similarity to Pick bodies, as seen on tau‐immunostaining (Figure 3 **a**), were only present within the cytoplasm of DG cells on LC3A immunostaining (Figure 3 **f**), though in some instances occasional structures resembling Pick bodies appeared also to be immunostained for LAMP‐2 (Figure 3 **c**).

### Semi‐quantitative analysis

#### LAMP‐1

**Figure 4:**
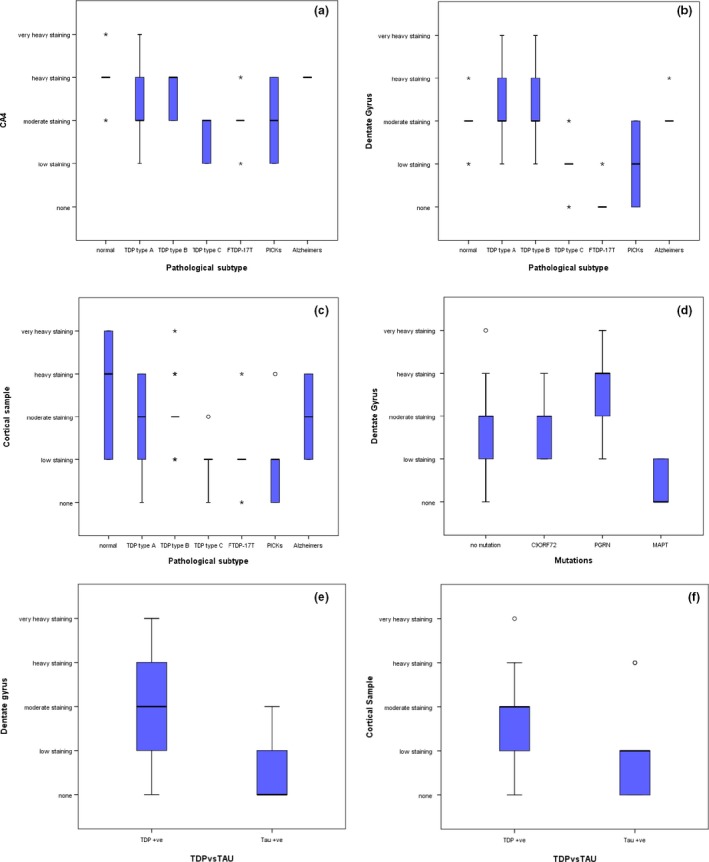
Box plots showing semi‐quantitative scores for rating of immunostaining for LAMP‐1. Scores for all histological groups (normal, FTLD‐TDP types **a**,** b** and **c**, FTLD‐tau associated with *MAPT* mutations (FTDP‐17T) and Pick bodies (PICKS) and Alzheimer's disease) are presented for the CA4 (**a**) and dentate gyrus (**b**) of the hippocampus and temporal cortex (**c**). Also shown are comparisons between FTLD genetic groups (no mutation, *C9ORF72*,* PGRN* and *MAPT* groups) (**d**) and between all FTLD‐TDP and FTLD‐tau cases for the dentate gyrus (**e**) and temporal cortex (**f**).

Scores for LAMP‐1 immunostaining in FTLD (collectively), AD and healthy controls analysed by Kruskal–Wallis were significantly different in area CA4 of the hippocampus (*H* = 8.5, *P* = 0.014), but not so in the DG (*H* = 0.81, *P* = 0.665) or Tcx (*H* = 3.0, *P* = 0.221). Post hoc testing for the CA4 region showed scores for the FTLD group were significantly less than those of the AD group (*P* = 0.017) but were not significantly different from controls (*P* = 0.060), which in turn did not differ from the AD group scores (*P* = 1.00). However, FTLD pathological subgroup analysis (Figure 4) revealed significant differences in scores for neuronal LAMP‐1 immunostaining between the five FTLD histological groups (i.e. FTLD‐TDP types A, B and C, FTLD‐tau with *MAPT* mutation or Pick bodies) for both the CA4 (*H* = 11.3, *P* = 0.024) (Figure 4 **a**) and DG (*H* = 24.7, *P* < 0.001) (Figure 4 **b**) regions of the hippocampus, and the Tcx (*H* = 14.8, *P* = 0.005) (Figure 4 **c**). In the CA4 region, this difference was driven by significantly lower LAMP‐1 scores in the FTLD‐TDP type C group compared to the FTLD‐TDP type A (*P* = 0.010) and FTLD‐TDP type B (*P* = 0.003) groups (Figure 4 **a**), but these were not significantly different from those of either of the FTLD‐tau groups. Similar findings were seen in the DG where LAMP‐1 scores in the FTLD‐TDP type C group were significantly lower than those in the FTLD‐TDP type A (*P* = 0.003) and FTLD‐TDP type B (*P* = 0.012) groups (Figure 4 **b**), but these were not significantly different from those of either of the FTLD‐tau groups. Here, scores for FTLD‐tau group due to *MAPT* mutation were significantly lower than those of the FTLD‐type A (*P* = 0.001) and type B (*P* < 0.001) groups (Figure 3 **b**), as were those for the FTLD‐tau group with Pick bodies (*P* = 0.004 and 0.017, respectively). In the Tcx, LAMP‐1 scores in the FTLD‐TDP type C group were significantly lower than those in the FTLD‐TDP type A (*P* = 0.022) and FTLD‐TDP type B (*P* = 0.002) groups, but these were not significantly different from those of either of the FTLD‐tau groups (Figure 4 **c**). LAMP‐1 scores in the FTLD‐tau *MAPT* group were also less than those in the FTLD‐TDP type B group (*P* = 0.023) (Figure 4 **c**) and scores for FTLD‐tau with Pick bodies were significantly lower than those of FTLD‐TDP type A (*P* = 0.041) and type B (*P* = 0.014) (Figure 4 **c**). Significantly lower levels of LAMP‐1 immunostaining were present in all cases of FTLD‐tau combined compared to those with FTLD‐TDP in the DG (*P* < 0.001) (Figure 3 **e**) and Tcx (*P* = 0.013) (Figure 4 **f**), but not in the CA4 region (*P* = 0.102).

A significant difference in scores for neuronal LAMP‐1 immunostaining between the four genetic groups was seen in the DG (*H* = 15.4, *P* = 0.001) (Figure 4 **d**) but not in the CA4 region (*H* = 4.7, *P* = 0.197) or in the Tcx (*H* = 4.0, *P* = 0.261). In the DG, this difference was driven by *C9orf72* (*P* = 0.001), *GRN* (*P* = 0.002) and FTLD no mutation groups (*P* = 0.005) all being greater than the *MAPT* group (Figure 4 **d**). LAMP‐1 scores in the *GRN* group were significantly higher than those in the nonmutation group (*P* = 0.022) and marginally higher than the *C9orf72* group (*P* = 0.044) (Figure 4 **d**).

#### LAMP‐2

There were no significant differences in the scores for LAMP‐2 immunostaining in FTLD (collectively), AD and controls for area CA4 of the hippocampus (*H* = 5.2, *P* = 0.073), DG (*H* = 0.83, *P* = 0.661) or Tcx (*H* = 2.1, *P* = 0.341). Similarly, no significant differences were found in scores for LAMP‐2 between the five FTLD histological subtypes (types A, B and C, FTLD‐Tau and Picks disease) for area CA4 of the hippocampus (*H* = 4.1, *P* = 0.400), DG (*H* = 4.9, *P* = 0.296) or Tcx (*H* = 4.5, *P* = 0.339). Likewise, there were no significant differences between all FTLD‐TDP and FTLD‐tau cases (*P* = 0.344 for CA4, *P* = 0.406 for DG and *P* = 0.705 for Tcx). FTLD‐tau and FTLD‐Picks cases also did not differ significantly (*P* = 0.792 for CA4, *P* = 0.792 for DG and *P* = 0.937 for Tcx). Comparisons of scores for FTLD cases bearing mutations in *C9orf72*,* GRN* and *MAPT*, and nonmutants were not significantly different for area CA4 of the hippocampus (*H* = 1.58, *P* = 0.665), DG (*H* = 1.54, *P* = 0.673) or Tcx (*H* = 1.05, *P* = 0.788).

#### Cathepsin D

There were no significant differences in the scores for CTSD immunostaining in FTLD (collectively), AD and healthy controls for area CA4 of the hippocampus (*H* = 3.5, *P* = 0.170), DG (*H* = 0.28, *P* = 0.869) or Tcx (*H* = 0.10, *P* = 0.949). No significant differences in the scores were found between the five FTLD histological subtypes for area CA4 of the hippocampus (*H* = 2.2, *P* = 0.698) or Tcx (*H* = 5.1, *P* = 0.275), though scores for the DG did differ significantly (*H* = 11.4, *P* = 0.022) with scores for FTLD‐TDP type B cases being significantly lower than those for either type A (*P* = 0.006) or type C (*P* = 0.020) but not different from all other groups, which in turn did not differ significantly. There were no significant differences between all FTLD‐TDP and FTLD‐tau cases (*P* = 0.323 for the CA4, *P* = 0.763 for the DG and *P* = 0.396 for the Tcx). FTLD‐tau and FTLD‐Pick's cases also did not differ significantly (*P* = 0.490 for the CA4, *P* = 0.275 for the DG and *P* = 0.778 for the Tcx). Comparisons of the scores for FTLD cases bearing mutations in *C9orf72*,* GRN* and *MAPT*, and nonmutants were not significantly different for area CA4 of the hippocampus (*H* = 0.61, *P* = 0.896), DG (*H* = 2.34, *P* = 0.504) or Tcx (*H* = 1.09, *P* = 0.780).

Hence, the present data indicate that in general there are no significant differences in CTSD immunoreactivity between any of the FTLD histological or genetic subgroups, except that scores in the FTLD‐TDP type B group were higher than those in type C but lower than in the type A subgroups.

#### EEA‐1

There was a significant difference between the scores for EEA‐1 immunostaining in FTLD (collectively), AD and healthy controls for area CA4 of the hippocampus (*H* = 7.7, *P* = 0.021), DG (*H* = 10.9, *P* = 0.004) or Tcx (*H* = 8.4, *P* = 0.015), with the scores for FTLD cases being significantly higher than those for control cases in all the three regions (CA4, *P* = 0.027; DG, *P* = 0.014 and Tcx, *P* = 0.015), but these did not differ from AD cases, which in turn did not differ from controls.

However, no significant differences in the scores were found between the five FTLD histological subtypes for areas CA4 (*H* = 4.7, *P* = 0.323) and DG (*H* = 4.5, *P* = 0.349) of the hippocampus and Tcx (*H* = 5.08, *P* = 0.037). Likewise, there were no significant differences between all FTLD‐TDP and FTLD‐tau cases (*P* = 0.408 for CA4, *P* = 0.666 for DG and *P* = 0.984 for Tcx). FTLD‐tau and FTLD‐Pick's cases also did not differ significantly (*P* = 0.224 for CA4, *P* = 0.862 for DG and *P* = 0.964 for Tcx). Comparisons of the scores for FTLD cases bearing mutations in *C9orf72*,* GRN* and *MAPT*, and nonmutants were not significantly different for area CA4 of the hippocampus (*H* = 4.55, *P* = 0.208), DG (*H* = 2.04, *P* = 0.564) or Tcx (*H* = 0.70, *P* = 0.874).

The present data indicate that although levels of EEA‐1 are higher than in controls, there are no significant differences in EEA‐1 immunoreactivity between any of the FTLD histological or genetic groups.

#### LC3A

Semi‐quantitative analysis revealed no significant difference in the scores for neuronal LC3A immunostaining between FTLD (collectively), AD and controls (DG, *H* = 0.39, *P* = 0.820; CA4, *H* = 0.94, *P* = 0.626; Tcx, *H* = 2.6, *P* = 0.271), or between any of the five FTLD histological (DG, *H* = 7.3, *P* = 0.119; CA4, *H* = 1.9, *P* = 0.753; Tcx, *H* = 7.7, *P* = 0.103) or four genetic (DG, *H* = 0.147, *P* = 0.986; CA4, *H* = 0.790, *P* = 0.852; Tcx, *H* = 1.86, *P* = 0.602) groups. There were also no significant differences in LC3A immunostaining in the DG (*P* = 0.194), CA4 (*P* = 0.927) or Tcx (*P* = 0.109) in all the cases of FTLD‐tau combined compared to those with FTLD‐TDP.

Hence, the present data indicate that there are no significant differences in LC3A immunoreactivity between any of the FTLD histological or genetic subgroups.

### Comparisons between FTLD‐TDP type A cases

Because there was a significant increase in LAMP‐1 immunostaining in FTLD‐TDP cases associated with *GRN* mutation compared to cases with mutations in *MAPT*, expansions in *C9orf72* or nonmutational cases (see earlier), and because seven of the cases with *C9orf72* expansions and five nonmutational cases share similar FTLD‐TDP type A pathology with the 12 *GRN* cases, we performed additional analyses in order to determine whether the increases in LAMP‐1 seen in *GRN* cases reflected the presence of *GRN* mutation *per se*, or were indicative of the underlying histological type regardless of the presence or type of mutation. Semi‐quantitative analysis revealed no significant differences in the scores between *GRN*,* C9orf72* or nonmutational cases with type A histology for LAMP‐1 (DG, *H* = 2.1, *P* = 0.341; CA4, *H* = 3.6, *P* = 0.168; Tcx, *H* = 1.7, *P* = 0.427), LAMP‐2 (DG, *H* = 4.9, *P* = 0.089; CA4, *H* = 2.0, *P* = 0.370; Tcx, *H* = 0.7, *P* = 0.711), CTSD (DG, *H* = 1.1, *P* = 0.565; CA4, *H* = 0.09, *P* = 0.954; Tcx, *H* = 1.0, *P* = 0.611), EEA‐1 (DG, *H* = 3.5, *P* = 0.175; CA4, *H* = 2.5, *P* = 0.288; Tcx, *H* = 2.2, *P* = 0.349) or LC3A (DG, *H* = 0.9, *P* = 0.643; CA4, *H* = 0.3, *P* = 0.859; Tcx, *H* = 2.9, *P* = 0.234). This suggests that the increases in LAMP‐1 immunostaining seen in *GRN* cases may be a property of the underlying type A histology rather than possession of *GRN* mutation *per se*.

### Correlations

In this way, significant positive correlations were seen for all FTLD cases combined between scores for LAMP‐2 and CTSD in the DG (*P* < 0.001) and Tcx (*P* < 0.001). In the FTLD‐TDP cases alone (all three histological subtypes combined), significant correlations were found between LAMP‐2 and CTSD in the CA4 (*P* < 0.001), DG (*P* = 0.002) and Tcx (*P* = 0.002), and for CTSD and EEA‐1 (*P* = 0.003) in the CA4 region alone. There were no significant correlations between any antibody combinations in any region for all FTLD‐tau cases combined. Further stratification into each of the five individual histological groups did not yield any consistent or significant associations.

Correlations were also performed when FTLD cases were stratified according the genetics. Significant correlations were found between LAMP‐2 and CTSD in the CA4 (*P* = 0.004), DG (*P* = 0.003) and Tcx (*P* = 0.007) in *MAPT* carriers, and between LAMP‐2 and CTSD in the CA4 (*P* = 0.009) in bearers of *C9orf72* expansion. There were no other significant correlations between any antibody combinations in any region in each of the genetic groups.

## Discussion

In the present study, we have investigated the cellular distribution and degree of immunostaining of five proteins, LAMP‐1, LAMP‐2, CTSD, EEA‐1 and LC3A, involved in the autophagosomal/lysosomal degradative pathways in patients with different histological and genetic forms of FTLD. The main findings can be summarized:

- There was less LAMP‐1 immunostaining in FTLD as a whole, compared to AD and control subjects, this being driven partly by loss of LAMP‐1 from patients with SD and FTLD‐TDP type C histology compared to those with FTLD‐TDP types A and B.
- Levels of LAMP‐1 immunostaining were also lower in FTLD‐tau as a whole than in FTLD‐TDP as a whole, despite the decreased levels of immunostaining in the FTLD‐TDP type C cases which would tend to reduce the magnitude of the overall group differences.
- LAMP‐2, CTSD, EEA‐1 and LC3A demonstrated normal patterns and intensity of immunostaining across all the pathological and genetic FTLD groups.
- LAMP‐2, CTSD and EEA‐1 immunostaining was normal in cases of FTLD‐TDP type C despite there being reduced LAMP‐1 immunostaining.
- LAMP‐1, but not LAMP‐2, CTSD, EEA‐1, was elevated in *GRN* mutants relative to other FTLD genetic and non‐genetic groups.
- LAMP‐1, LAMP‐2, CTSD, EEA‐1 and LC3A proteins were all localized within Pick bodies.

As pointed out above, the loss of LAMP‐1 immunostaining in FTLD as a whole, compared to AD and controls, appeared to be driven in part by loss of LAMP‐1 from patients with SD and FTLD‐type C pathology. It might be argued that this is simply a reflection of the particularly severe neurodegeneration of the temporal lobe resulting in neuronal atrophy and loss occurring in this form of FTLD 1. However, if this were so, then similar losses of the other four ALP proteins, and correlations between LAMP‐1 levels and the levels of the other four protein markers, would have been anticipated. That this was not seen argues for loss of LAMP‐1 in FTLD‐TDP type C to be a specific aspect of the pathogenesis, and one that is not shared by the other FTLD‐TDP histological subtypes. Such a loss of LAMP‐1 protein in FTLD‐TDP type C cases suggests a selective reduction in the number of lysosomes available for protein degradation, while maintenance of LAMP‐2, CTSD, EEA‐1 proteins in the face of this loss in LAMP‐1 would imply that functional capacity of residual lysosomes is maintained or even upregulated to compensate for a shortfall in lysosomal number *per se*. Nonetheless, it remains unclear what initiating factors or processes might be driving loss of LAMP‐1 in this form of FTLD.

The preservation of LAMP‐1 in FTLD‐TDP types A and B, and in the genetic forms associated with these histological subtypes (i.e. *C9orf72* expansions and *GRN* mutations), suggests maintenance of lysosomal structure. Maintenance of LAMP‐2, CTSD and EEA‐1 levels imply lysosomal function is also preserved. Normal LC3A immunostaining indicates autophagosomal function is unchanged. Hence, despite evidence that both C9orf72 28, 29, 30 and PGRN 26, 27 proteins play a role in lysosomal‐mediated degradation, this does not seem to be reflected by losses in any of the proteins involved in lysosomal/autophagosomal function in patients bearing expansions in *C9orf72* or mutations in *GRN*. Indeed, we noted that levels of LAMP‐1 staining were particularly high in patients with *GRN* mutations relative to other genetic and non‐genetic forms of FTLD, suggesting that an upregulation of lysosomal number/function, in response to lack of PGRN, might underlie this form of FTLD. This latter observation is consistent with findings of upregulated LAMP‐1 in PGRN deficient mice 33. Given the development of NCL in *GRN* double knockouts 33, elevated LAMP‐1 levels in *GRN* mutation carriers may indicate an increased number of dysfunctional lysosomes. There is evidence that *GRN* is part of a network of lysosomal genes that is upregulated in response to transcription factor EB (TFEB). When PGRN levels are reduced, for example in *GRN* knock outs, TFEB is activated leading to elevated lysosomal biogenesis that includes the transcription of LAMP‐1 47 and elevation of CTSD 48. Despite these observations that CTSD activity is regulated by PGRN 27, 48, 49, we did not observe any shortfall in CTSD in neurones in *GRN* carriers. However, it is very difficult to assess lysosomal/autophagosomal functionality with immunostaining alone, which can only define relative levels of autophagosomes and lysosomes present within cells and is subject to ‘ceiling’ or ‘floor’ effects. Nonetheless, in a recent study 49, high levels of CTSD were also noted in neurones of the cerebral cortex bearing TDP‐43 aggregates in *GRN* mutation carriers supporting present observations.

The reduction in LAMP‐1 in patients with FTLD, overall, also seems to be driven in part by a loss of activity in patients with FTLD‐tau due to *MAPT* mutation and those with FTLD‐tau associated with Pick bodies. Consistent levels of LAMP‐2, CTSD and EEA‐1 across all the FTLD subgroups suggests that CMA‐related pathological changes in LAMP‐1 in patients with FTLD‐tau are not due to alterations in absolute protein levels. There are other possible reasons for this. In cell culture, tau has been shown to be cleared via deliberate exocytosis 50. Lant *et al*. 51 reported an increased microglial activity in the temporal cortex and hippocampus in the same cases bearing *MAPT* mutation as those included in the present study, suggesting tau may be exocytosed and degraded by surrounding glia. Such a mechanism would bypass lysosomal degradation explaining the low LAMP‐1 levels. Polito *et al*. 52 showed that induction of TFEB exacerbates tau pathology in part due to increased lysosomal biogenesis, implying that in FTLD‐tau lysosomal clearance may be deficient. This is supported by present observations of normal autophagosome (LC3A) activity. The *MAPT* mutation cases used in this study were all bore exon 10+16 mutation which results in the production of more 4R tau isoforms 3. Imbalances in tau isoform ratio promote microtubule dysfunction. Kochl *et al*. 53 demonstrated that microtubules are essential for autophagolysosome fusion. Collectively, these observations suggest that changes in tau leading to hyperphosphorylation and microtubule dysfunction may play a role in the pathogenesis of FTLD‐tau by preventing normal lysosomal fusion and subsequent degradation of autophagosomes. The present findings of similar changes in LAMP‐1 in the non‐genetic cases of FTLD‐tau with Pick bodies, which are characterized by accumulation of 3‐repeat tau, points to a shared pathogenesis irrespective of what particular tau isoforms might be accumulated. Tau has been shown to disrupt lysosomal membrane integrity, since when it undergoes CMA, highly amyloidogenic tau fragments are cleaved resulting in lysosomal membrane permeabilization 54. Hence, the tau aggregates in FTLD‐tau could be the cause of the lysosomal deficiency, rather than a consequence thereof.

The oval inclusion bodies seen on LC3A, but only infrequently on LAMP‐2, and not at all on LAMP‐1, CTSD and EEA‐1, immunostaining in DG in those cases of FTLD‐tau associated with Pick bodies (Pick's disease) are likely to be Pick bodies, since these are well known to cluster in neurones of the dentate gyrus, CA1 and pyramidal neurones of the frontal and temporal cortex 55. Other studies have shown that Pick bodies, from an early stage, contain LC3A‐binding proteins such as p62 24, 56 and ubiquitin 56 along with tau proteins. This colocalization of autophagy related proteins may reflect an attempt to degrade Pick bodies along this pathway. If so, the lack of immunostaining of these structures with LAMP‐1 suggests that lysosomes may not be clearing autophagosomes effectively and that autophagosomes are sequestered into Pick bodies, thereby preventing their transport to the lysosome for degradation. If so, this could explain why only proteins that bind specifically to LC3A are present in Pick bodies, since LAMP‐1 is not one of these binding partners. However, having said that others have observed that NBR1, another binding partner of LC3A, is not present in Pick bodies 57, and Miki *et al*. 58 have shown that HDAC6, which deacetylates the contactin necessary for fusion of the autophagosome to the lysosome, is also absent. Hence, it is possible that formation of Pick bodies, in contrast to Lewy bodies where all relevant markers of the autophagosome/lysosome system, such as p62, ubiquitin, LC3A, NBR1 and HDAC6 17, 24, 57, 58 are present, is not directly related to the autophagosome/lysosome system and that marker proteins of this are simply sequestered passively into these along with other unrelated proteins.

In conclusion, the present study provides further evidence that changes in lysosomal structure or function underpin the pathogenesis of neurodegenerative disorders such as FTLD. The lower LAMP‐1 levels in FTLD‐tau suggest that the underlying pathological mechanism, involving a failure to clear tau aggregates, may lie with a relative deficiency of lysosomes, or defective vesicular transport. The relatively preserved levels of LAMP‐1 in FTLD‐TDP (types A and B), or even elevated levels in *GRN* mutation carriers, suggests the failure to clear TDP‐43 aggregates may lie with lysosomal dysfunction rather than a lack of available lysosomes or degradative enzymes.

## Author's contributions

Hamish Bain helped design the study, performed most of the immunohistochemical staining, did all the semiquantitative assessments and assisted with writing the paper.

Yvonne Davidson provided technical assistance and performed the other immunohistochemical staining.

Sarah Ryan performed the double immunofluorescence work.

Andrew Robinson prepared the wax sections and performed all the statistical analyses.

Sara Rollinson and Stuart Pickering‐Brown performed the genetic analyses.

Anna Richardson, Matthew Jones and Julie Snowden clinically characterized the patients.

David Mann designed the study and wrote the paper.

## Conflict of interest

The authors declare that they have no Conflicts of Interest.

## Ethical agreements

This work was approved by Manchester Brain Bank under its devolved Generic Tissue Bank Ethical Agreements conferred by Newcastle and North Tyneside 1 Research Ethics (09‐H0906‐52 +5).

## Supporting information


**Figure S1.** Representative images of LAMP‐1 immunostaining in the dentate gyrus illustrating the grading system employed for scoring levels of immunostaining.

**Figure S2.** Results of double immunofluorescence labelling of dentate gyrus granule cells in patient with FTLD‐TDP type B and expansion in *C9orf72* (case #9). Reading from left to right, panels show DAPI labelling of nuclei (blue), TDP‐43 immunofluorescence (green), LAMP‐1 immunofluorescence (red) and merge of all the three images. Red granules representing ALP structures seen in LAMP‐1 panel are not identified in TDP‐43 panel where only nuclei are visible. In the merged image, ALP structures remain red showing no colocalization with TDP‐43, whereas nuclei are seen to be cyan in colour due to the merging of DAPI and TDP‐43 fluorescence. Scale bar indicates 10 μm.

**Table S1.** Selected clinical, neuropathological and genetic details of cases employed in the study.

## References

[1] Snowden JS , Neary D , Mann DMA . Frontotemporal dementia. Lancet Neurol 2005; 4: 771–80 16239184 10.1016/S1474-4422(05)70223-4

[2] Saxon JA , Harris JM , Thompson JC , Jones M , Richardson AMT , Langheinrich T , Neary D , Mann DMA , Snowden JS . Semantic dementia, progressive non‐fluent aphasia and their association with amyotrophic lateral sclerosis. J Neurol Neurosurg Psychiatry 2017; 88: 711–712. 28554960 10.1136/jnnp-2016-314912 PMC5537533

[3] Hutton M , Lendon CL , Rizzu P , Baker M , Froelich S , Houlden M , Pickering‐Brown SM , Chakraverty S , Isaacs A , Grover A , Hackett J , Adamson J , Lincoln S , Dickson D , Davies P , Petersen RC , Stevens M , de Graaf E , Wauters E , van Baren J , Hillebrand M , Joosse M , Kwon JM , Nowotny P , Che LK , Norton J , Morris JC , Reed LA , Trojanowski JQ , Basun H , Lannfelt L , Neystat M , Fahn S , Dark F , Tannenberg T , Dodd P , Hayward N , Kwok JBJ , Schofield PR , Andreadis A , Snowden J , Craufurd D , Neary D , Owen F , Oostra BA , Hardy J , Goate A , van Swieten J , Mann DM , Lynch T , Heutink P . Association of missense and 5'–splice‐site mutation in tau with inherited dementia FTDP‐17. Nature 1998; 393: 702–5 9641683 10.1038/31508

[4] Baker M , Mackenzie IRA , Pickering‐Brown SM , Gass J , Rademakers R , Lindholm C , Snowden J , Adamson J , Sadovnick AD , Rollinson S , Cannon A , Dwosh E , Neary D , Melquist S , Richardson A , Dickson D , Eriksen J , Robinson T , Zehr C , Dickey CA , Crook R , McGowan E , Mann D , Boeve B , Feldman H , Hutton M . Mutations in progranulin cause tau‐negative frontotemporal dementia linked to chromosome 17. Nature 2006; 442: 916–9 16862116 10.1038/nature05016

[5] Cruts M , Gijselinck I , van der Zee J , Engelborghs S , Wils H , Pirici D , Rademakers R , Vandenberghe R , Dermaut B , Martin JJ , van Duijn C , Peeters K , Sciot R , Santens P , De Pooter T , Mattheijssens M , Van den Broeck M , Cuijt I , Vennekens K , De Deyn PP , Kumar‐Singh S , Van Broeckhoven C . Null mutations in progranulin cause ubiquitin‐positive frontotemporal dementia linked to chromosome 17q21. Nature 2006; 442: 920–4 16862115 10.1038/nature05017

[6] DeJesus‐Hernandez M , Mackenzie IR , Boeve BF , Boxer AL , Baker M , Rutherford NJ , Nicholson AM , Finch NA , Flynn H , Adamson J , Kouri N , Wojtas A , Sengdy P , Hsiung GY , Karydas A , Seeley WW , Josephs KA , Coppola G , Geschwind DH , Wszolek ZK , Feldman H , Knopman DS , Petersen RC , Miller BL , Dickson DW , Boylan KB , Graff‐Radford NR , Rademakers R . Expanded GGGGCC hexanucleotide repeat in noncoding region of C9ORF72 causes chromosome 9p‐linked FTD and ALS. Neuron 2011; 72: 245–56 21944778 10.1016/j.neuron.2011.09.011 PMC3202986

[7] Renton AE , Majounie E , Waite A , Simón‐Sánchez J , Rollinson S , Gibbs JR , Laaksovirta H , Schymick JC , van Swieten J , Myllykangas L , Kalimo H , Paetau A , Abramzon Y , Remes AM , Kaganovich A , Scholz SW , Duckworth J , Ding J , Harmer DW , Hernandez DG , Johnson JO , Mok K , Ryten M , Trabzuni D , Guerreiro RJ , Orrell RW , Neal J , Murray A , Pearson J , Jansen IE , Sondervan D , Seelaar H , Blake D , Young K , Halliwell N , Callister J , Toulson G , Richardson A , Gerhard A , Snowden J , Mann D , Neary D , Nalls MA , Peuralinna T , Jansson L , Isoviita V‐M , Kaivorinne A‐L , Holtta‐Vuori M , Ikonen E , Sulkava R , Benatar M , Wuu J , Chio A , Restagno G , Borghero G , Sabatelli M ; The ITALSGEN Consortium , Heckerman D , Rogaeva E , Zinman L , Rothstein J , Sendtner M , Drepper C , Eichler EE , Alkan C , Abdullaev Z , Pack SD , Dutra A , Pak E , Hardy J , Singleton A , Williams NM , Heutink P , Pickering‐Brown S , Morris HR , Tienari PJ , Traynor BJ . A hexanucleotide repeat expansion in C9ORF72 is the cause of chromosome 9p21‐linked ALS‐FTD. Neuron 2011; 72: 257–68. 21944779 10.1016/j.neuron.2011.09.010 PMC3200438

[8] Watts GD , Wymer J , Kovach MJ , Mehta SG , Mumm S , Darvish D , Pestronk A , Whyte MP , Kimonis VE . Inclusion body myopathy associated with Paget disease of bone and frontotemporal dementia is caused by mutant valosin‐containing protein. Nature Genet 2004; 36: 377–81 15034582 10.1038/ng1332

[9] Skibinski G , Parkinson NJ , Brown JM , Chakrabarti L , Lloyd SL , Hummerich H , Nielsen JE , Hodges JR , Spillantini MG , Thusgaard T , Brandner S , Brun A , Rossor MN , Gade A , Johannsen P , Sørensen SA , Gydesen S , Fisher EM , Collinge J . Mutations in the endosomal ESCRTIII‐complex subunit CHMP2B in frontotemporal dementia. Nature Genet 2005; 37: 806–8 16041373 10.1038/ng1609

[10] Deng HX , Chen W , Hong ST , Boycott KM , Gorrie GH , Siddique N , Yang Y , Fecto F , Shi Y , Zhai H , Jiang H , Hirano M , Rampersaud E , Jansen GH , Donkervoort S , Bigio EH , Brooks BR , Ajroud K , Sufit RL , Haines JL , Mugnaini E , Pericak‐Vance MA , Siddique T . Mutations in UBQLN2 cause dominant X‐linked juvenile and adult‐onset ALS and ALS/dementia. Nature 2011; 477: 211–5 21857683 10.1038/nature10353 PMC3169705

[11] Maruyama H , Morino H , Ito H , Izumi Y , Kato H , Watanabe Y , Kinoshita Y , Kamada M , Nodera H , Suzuki H , Komure O , Matsuura S , Kobatake K , Morimoto N , Abe K , Suzuki N , Aoki M , Kawata A , Hirai T , Kato T , Ogasawara K , Hirano A , Takumi T , Kusaka H , Hagiwara K , Kaji R , Kawakami H . Mutations of optineurin in amyotrophic lateral sclerosis. Nature 2010; 465: 223–6 20428114 10.1038/nature08971

[12] Miller L , Rollinson S , Callister J , Young K , Harris J , Gerhard A , Richardson A , Jones M , Snowden J , Mann DMA , Pickering‐Brown SM . p62/SQSTM1 analysis in frontotemporal lobar degeneration. Neurobiol Aging 2015; 36: e5–9 10.1016/j.neurobiolaging.2014.08.035 25433461

[13] Van Deerlin VM , Sleiman PM , Martinez‐Lage M , Chen‐Plotkin A , Wang LS , Graff‐Radford NR , Dickson DW , Rademakers R , Boeve BF , Grossman M , Arnold SE , Mann DM , Pickering‐Brown SM , Seelaar H , Heutink P , van Swieten JC , Murrell JR , Ghetti B , Spina S , Grafman J , Hodges J , Spillantini MG , Gilman S , Lieberman AP , Kaye JA , Woltjer RL , Bigio EH , Mesulam M , Al‐Sarraj S , Troakes C , Rosenberg RN , White CL 3rd , Ferrer I , Lladó A , Neumann M , Kretzschmar HA , Hulette CM , Welsh‐Bohmer KA , Miller BL , Alzualde A , de Lopez Munain A , McKee AC , Gearing M , Levey AI , Lah JJ , Hardy J , Rohrer JD , Lashley T , Mackenzie IR , Feldman HH , Hamilton RL , Dekosky ST , der van Zee J , Kumar‐Singh S , Van Broeckhoven C , Mayeux R , Vonsattel JP , Troncoso JC , Kril JJ , Kwok JB , Halliday GM , Bird TD , Ince PG , Shaw PJ , Cairns NJ , Morris JC , McLean CA , DeCarli C , Ellis WG , Freeman SH , Frosch MP , Growdon JH , Perl DP , Sano M , Bennett DA , Schneider JA , Beach TG , Reiman EM , Woodruff BK , Cummings J , Vinters HV , Miller CA , Chui HC , Alafuzoff I , Hartikainen P , Seilhean D , Galasko D , Masliah E , Cotman CW , Tuñón MT , Martínez MC , Munoz DG , Carroll SL , Marson D , Riederer PF , Bogdanovic N , Schellenberg GD , Hakonarson H , Trojanowski JQ , Lee VM . Common variants at 7p21 are associated with frontotemporal lobar degeneration with TDP‐43 inclusions. Nat Genet 2010; 42: 234–9 20154673 10.1038/ng.536 PMC2828525

[14] Shi J , Shaw CL , Richardson AMT , Bailey K , Tian J , Varma AR , Neary D , Snowden JS , Mann DMA . Histopathological changes underlying frontotemporal lobar degeneration with clinicopathological correlation. Acta Neuropathol 2005; 110: 501–12 16222525 10.1007/s00401-005-1079-4

[15] Chakrabarti A , Chen AW , Varner JD . A review of the mammalian unfolded protein response. Biotechnol Bioeng 2011; 108: 2777–93 21809331 10.1002/bit.23282 PMC3193940

[16] Kroemer G , Mariño G , Levine B . Autophagy and the integrated stress response. Mol Cell 2010; 40: 280–93 20965422 10.1016/j.molcel.2010.09.023 PMC3127250

[17] Kuzuhara S , Mori H , Izumiyama N , Yoshimura M , Ihara Y . Lewy bodies are ubiquitinated. Acta Neuropathol 1988; 75: 345–53 3364159 10.1007/BF00687787

[18] Kitada T , Asakawa S , Hattori N , Matsumine H , Yamamura Y , Minoshima S , Yokochi M , Mizuno Y , Shimizu N . Mutations in the parkin gene cause autosomal recessive juvenile parkinsonism. Nature 1998; 39: 605–8 10.1038/33416 9560156

[19] Leroy E , Boyer R , Auburger G , Leube B , Ulm G , Mezey E , Harta G , Brownstein MJ , Jonnalagada S , Chernova T , Dehejia A , Lavedan C , Gasser T , Steinbach PJ , Wilkinson KD , Polymeropoulos MH . The ubiquitin pathway in Parkinson's disease. Nature 1998; 395: 451–2 9774100 10.1038/26652

[20] McNaught K , Belizaire R , Isacson O , Jenner P , Olanow CW . Altered proteasomal function in sporadic Parkinson's disease. Expl Neurol 2003; 179: 38–46 10.1006/exnr.2002.8050 12504866

[21] Bukhatwa S , Zeng B‐Y , Rose S , Jenner P . A comparison of changes in proteasomal subunit expression in the substantia nigra in Parkinson's disease, multiple system atrophy and progressive supranuclear palsy. Brain Res 2010; 1326: 174–83 20176003 10.1016/j.brainres.2010.02.045

[22] Dai RM , Li CC . Valosin‐containing protein is a multi‐ubiquitin chain‐targeting factor required in ubiquitin‐proteasome degradation. Nat Cell Biol 2001; 3: 740–4 11483959 10.1038/35087056

[23] Markovinovic A , Cimbro R , Ljutic T , Kriz J , Rogelj B , Munitic I . Optineurin in amyotrophic lateral sclerosis: multifunctional adaptor protein at the crossroads of different neuroprotective mechanisms. Prog Neurobiol 2017; 154: 1–20 28456633 10.1016/j.pneurobio.2017.04.005

[24] Kuusisto E , Salminen A , Alfuzoff I . Ubiquitin‐binding protein p62 is present in neuronal and glial inclusions in human tauopathies and synucleinopathies. NeuroReport 2001; 12: 2085–90 11447312 10.1097/00001756-200107200-00009

[25] Rothenberg C , Srinivasan D , Mah L , Kaushik S , Peterhoff CM , Ugolino J , Fang S , Cuervo AM , Nixon RA , Monteiro MJ . Ubiquilin functions in autophagy and is degraded by chaperone‐mediated autophagy. Hum Mol Genet 2010; 19: 3219–32 20529957 10.1093/hmg/ddq231 PMC2908472

[26] Hu F , Padukkavidana T , Vægter CB , Brady OA , Zheng Y , Mackenzie IR , Feldman HH , Nykjaer A , Strittmatter SM . Sortilin‐mediated endocytosis determines levels of the frontotemporal dementia protein, progranulin. Neuron 2010; 68: 654–67 21092856 10.1016/j.neuron.2010.09.034 PMC2990962

[27] Zhou X , Sun L , Bastos de Oliveira F , Qi X , Brown WJ , Smolka MB , Sun Y , Hu F . Prosaposin facilitates sortilin‐independent lysosomal trafficking of progranulin. J Cell Biol 2015; 210: 991–1002 26370502 10.1083/jcb.201502029 PMC4576858

[28] Sellier C , Campanari ML , Julie Corbier C , Gaucherot A , Kolb‐Cheynel I , Oulad‐Abdelghani M , Ruffenach F , Page A , Ciura S , Kabashi E , Charlet‐Berguerand N . Loss of C9ORF72 impairs autophagy and synergizes with polyQ Ataxin‐2 to induce motor neuron dysfunction and cell death. EMBO J 2016; 35: 1276–97 27103069 10.15252/embj.201593350 PMC4910533

[29] Sullivan PM , Zhou X , Robins AM , Paushter DH , Kim D , Smolka MB , Hu F . The ALS/FTLD associated protein C9orf72 associates with SMCR8 and WDR41 to regulate the autophagy‐lysosome pathway. Acta Neuropathol Commun 2016; 4: 5 27193190 10.1186/s40478-016-0324-5 PMC4870812

[30] Webster CP , Smith EF , Bauer CS , Moller A , Hautbergue GM , Ferraiuolo L , Myszczynska MA , Higginbottom A , Walsh MJ , Whitworth AJ , Kaspar BK , Meyer K , Shaw PJ , Grierson AJ , De Vos KJ . The C9orf72 protein interacts with Rab1a and the ULK1 complex to regulate initiation of autophagy. EMBO J 2016; 35: 1656–76 27334615 10.15252/embj.201694401 PMC4969571

[31] Smith KR , Damiano J , Franceschetti S , Carpenter S , Canafoglia L , Morbin M , Rossi G , Pareyson D , Mole SE , Staropoli JF , Sims KB , Lewis J , Lin WL , Dickson DW , Dahl HH , Bahlo M , Berkovic SF . Strikingly different clinicopathological phenotypes determined by progranulin‐mutation dosage. Am J Hum Genet 2012; 90: 1102–7 22608501 10.1016/j.ajhg.2012.04.021 PMC3370276

[32] Almeida MR , Macário MC , Ramos L , Baldeiras I , Ribeiro MH , Santana I . Portuguese family with the co‐occurrence of frontotemporal lobar degeneration and neuronal ceroid lipofuscinosis phenotypes due to progranulin gene mutation. Neurobiol Aging 2016; 41: e1–200.e5 10.1016/j.neurobiolaging.2016.02.019 27021778

[33] Götzl JK , Mori K , Damme M , Fellerer K , Tahirovic S , Kleinberger G , Janssens J , van der Zee J , Lang CM , Kremmer E , Martin JJ , Engelborghs S , Kretzschmar HA , Arzberger T , Van Broeckhoven C , Haass C , Capell A . Common pathobiochemical hallmarks of progranulin‐associated frontotemporal lobar degeneration and neuronal ceroid lipofuscinosis. Acta Neuropathol 2014; 127: 845–60 24619111 10.1007/s00401-014-1262-6

[34] Zhou X , Paushter DH , Feng T , Pardon CM , Mendoza CS , Hu F . Regulation of cathepsin D activity by the FTLD protein progranulin. Acta Neuropathol 2017; 134: 151–3 28493053 10.1007/s00401-017-1719-5 PMC5568051

[35] Eskelinen E‐L . Roles of LAMP‐1 and LAMP‐2 in lysosome biogeneis and autophagy. Mol Aspects Med 2006; 27: 495–502 16973206 10.1016/j.mam.2006.08.005

[36] Kaushik S , Cuervo AM . Chaperone‐mediated autophagy: a unique way to enter the lysosome world. Trend Cell Biol 2012; 22: 407–17 10.1016/j.tcb.2012.05.006 PMC3408550 22748206

[37] Vidoni C , Follo C , Savino M , Melone MAB , Isidoro C . The role of cathepsin D in the pathogenesis of human neurodegenerative diseases. Med Res Rev 2016; 36: 845–70 27114232 10.1002/med.21394

[38] Christoforidis S , McBride HM , Burgoyne RD , Zerial M . The Rab5 effector EEA1 is a core component of endosome docking. Nature 1999; 397: 621–5 10050856 10.1038/17618

[39] Neary D , Snowden JS , Gustafson L , Passant U , Stuss D , Black S , Freedman M , Kertesz A , Robert PH , Albert M , Boone K , Miller BL , Cummings J , Benson DF . Frontotemporal lobar degeneration: a consensus on clinical diagnostic criteria. Neurology 1998; 51: 1546–54 9855500 10.1212/wnl.51.6.1546

[40] Gorno‐Tempini ML , Hillis AE , Weintraub S , Kertesz A , Mendez M , Cappa SF , Ogar JM , Rohrer JD , Black S , Boeve BF , Manes F , Dronkers NF , Vandenberghe R , Rascovsky K , Patterson K , Miller BL , Knopman DS , Hodges JR , Mesulam MM , Grossman M . Classification of primary progressive aphasia and its variants. Neurology 2011; 76: 1006–14 21325651 10.1212/WNL.0b013e31821103e6 PMC3059138

[41] Rascovsky K , Hodges JR , Knopman D , Mendez MF , Kramer JH , Neuhaus J , van Swieten JC , Seelaar H , Dopper EG , Onyike CU , Hillis AE , Josephs KA , Boeve BF , Kertesz A , Seeley WW , Rankin KP , Johnson JK , Gorno‐Tempini ML , Rosen H , Prioleau‐Latham CE , Lee A , Kipps CM , Lillo P , Piguet O , Rohrer JD , Rossor MN , Warren JD , Fox NC , Galasko D , Salmon DP , Black SE , Mesulam M , Weintraub S , Dickerson BC , Diehl‐Schmid J , Pasquier F , Deramecourt V , Lebert F , Pijnenburg Y , Chow TW , Manes F , Grafman J , Cappa SF , Freedman M , Grossman M , Miller BL . Sensitivity of revised diagnostic criteria for the behavioural variant of frontotemporal dementia. Brain 2011; 134: 2456–71 21810890 10.1093/brain/awr179 PMC3170532

[42] Snowden JS , Thompson JC , Stopford CL , Richardson AMT , Gerhard A , Neary D , Mann DMA . The clinical diagnosis of early‐onset dementias: diagnostic accuracy and clinicopathological relationships. Brain 2012; 135: 693–708 21840888 10.1093/brain/awr189

[43] Thompson JC , Stopford CL , Snowden JS , Neary D . Qualitative neuropsychological performance characteristics in frontotemporal dementia and Alzheimer's disease. J Neurol Neurosurg Psychiatr 2005; 76: 920–7 10.1136/jnnp.2003.033779 PMC1739700 15965196

[44] Mackenzie IRA , Neumann M , Baborie A , Sampathu DM , Du Plessis D , Jaros E , Perry RH , Trojanowski JQ , Mann DMA , Lee VM‐Y . A harmonized classification system for FTLD‐TDP pathology. Acta Neuropathol 2011; 122: 111–3 21644037 10.1007/s00401-011-0845-8 PMC3285143

[45] Mann DMA , Rollinson S , Robinson A , Callister J , Snowden JS , Gendron T , Petrucelli L , Masuda‐Suzukake M , Hasegawa M , Davidson YS , Pickering‐Brown S . Dipeptide repeat proteins are present in the p62 positive inclusions in patients with frontotemporal lobar degeneration and motor neurone disease associated with expansions in C9ORF72. Acta Neuropathol Comm 2013; 1: 68 10.1186/2051-5960-1-68 PMC3893586 24252525

[46] Davidson Y , Barker H , Robinson AC , Troakes C , Smith B , Al‐Sarraj S , Shaw C , Rollinson S , Masuda‐Suzukake M , Hasegawa M , Pickering‐Brown S , Snowden JS , Mann DMA . Brain distribution of dipeptide repeat proteins in frontotemporal lobar degeneration and motor neurone disease associated with expansions in *C9ORF72* . Acta Neuropathol Comm 2014; 2: 70 10.1186/2051-5960-2-70 PMC4229740 24950788

[47] Sardiello M , Donaudy F , Embrione V , Polishchuk RS , Banfi S , Parenti G , Cattaneo E , Ballabio A . A gene network regulating lysosomal biogenesis and function. Science 2009; 325: 473–7 19556463 10.1126/science.1174447

[48] Beel S , Moisse M , Damme M , De Muynck L , Robberecht W , Van Den Bosch L , Saftig P , Van Damme P . Progranulin functions as a cathepsin D chaperone to stimulate axonal outgrowth in vivo. Hum Mol Genet 2017; 26: 2850–63 28453791 10.1093/hmg/ddx162 PMC5886064

[49] Tanaka Y , Suzuki G , Matsuwaki T , Hosokawa M , Serrano G , Beach TG , Yamanouchi K , Hasegawa M , Nishihara M . Progranulin regulates lysosomal function and biogenesis through acidification of lysosomes. Hum Mol Genet 2017; 26: 969–88 28073925 10.1093/hmg/ddx011

[50] Simon D , García‐García E , Royo F , Falcón‐Pérez JM , Avila J . Proteostasis of tau. Tau overexpression results in its secretion via membrane vesicles. FEBS Lett 2012; 586: 527–34 10.1016/j.febslet.2011.11.022 22138183

[51] Lant SB , Robinson AC , Thompson JC , Rollinson S , Pickering‐Brown S , Snowden JS , Davidson YS , Gerhard A , Mann DM . Patterns of microglial cell activation in frontotemporal lobar degeneration. Neuropathol Appl Neurobiol 2014; 40: 686–96 24117616 10.1111/nan.12092

[52] Polito VA , Li H , Martini‐Stoica H , Wang B , Yang L , Xu Y , Swartzlander DB , Palmieri M , di Ronza A , Lee VM , Sardiello M , Ballabio A , Zheng H . Selective clearance of aberrant tau proteins and rescue of neurotoxicity by transcription factor EB. EMBO Mol Med 2014; 6: 1142–60 25069841 10.15252/emmm.201303671 PMC4197862

[53] Kochl R , Hu XW , Chan EY , Tooze SA . Microtubules facilitate autophagosome formation and fusion of autophagosomes with endosomes. Traffic 2006; 7: 129–45 16420522 10.1111/j.1600-0854.2005.00368.x

[54] Wang Y , Martinez‐Vicente M , Krüger U , Kaushik S , Wong E , Mandelkow EM , Cuervo AM , Mandelkow E . Tau fragmentation, aggregation and clearance: the dual role of lysosomal processing. Hum Mol Genet 2009; 18: 4153–70 19654187 10.1093/hmg/ddp367 PMC2758146

[55] Armstrong RA , Cairns NJ , Lantos PL . Clustering of Pick bodies in the dentate gyrus in Pick's disease. Neuropathology 2000; 20: 170–5 11132931 10.1046/j.1440-1789.2000.00328.x

[56] Miki Y , Mori F , Tanji K , Kurotaki H , Kakita A , Takahashi H , Wakabayashi K . An autopsy case of incipient Pick's disease: immunohistochemical profile of early‐stage Pick body formation. Neuropathology 2014; 34: 386–91 24444359 10.1111/neup.12104

[57] Odagiri S , Tanji K , Mori F , Kakita A , Takahashi H , Wakabayashi K . Autophagic adapter protein NBR1 is localised to Lewy bodies and glial cytoplasmic inclusions and is involved in aggregate formation in α‐synucleinopathy. Acta Neuropathol 2012; 124: 173–86 22484440 10.1007/s00401-012-0975-7

[58] Miki Y , Mori F , Tanji K , Kakita A , Takahashi H , Wakabayashi K . Accumulation of histone deacetylase 6, an aggresome‐related protein, is specific to Lewy bodies and glial cytoplasmic inclusions. Neuropathology 2011; 31: 561–8 21284752 10.1111/j.1440-1789.2011.01200.x

