# Supplementary figures and images for: The role of lysosomes and autophagosomes in frontotemporal lobar degeneration
Source: Neuropathol Appl Neurobiol. 2018 Jun 19;45(3):244–61. doi: 10.1111/nan.12500 (PMC6487817; doi:10.1111/nan.12500)

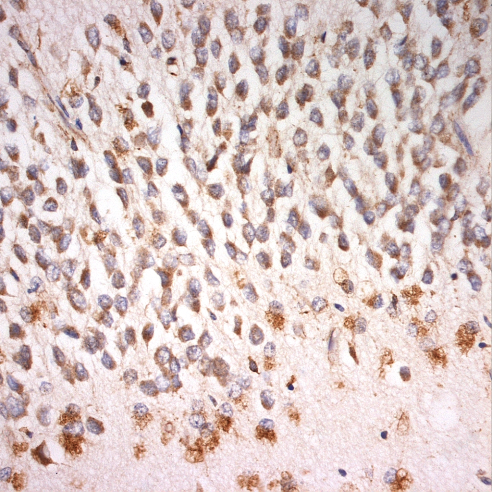

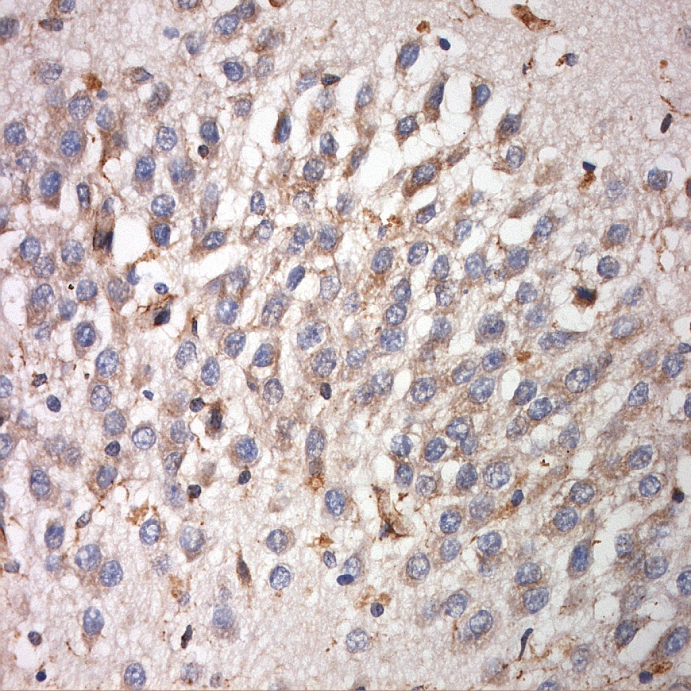

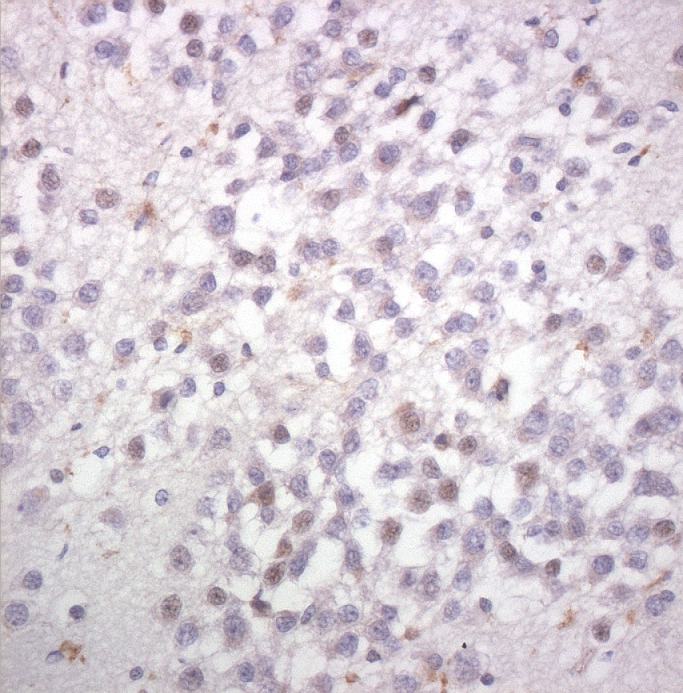

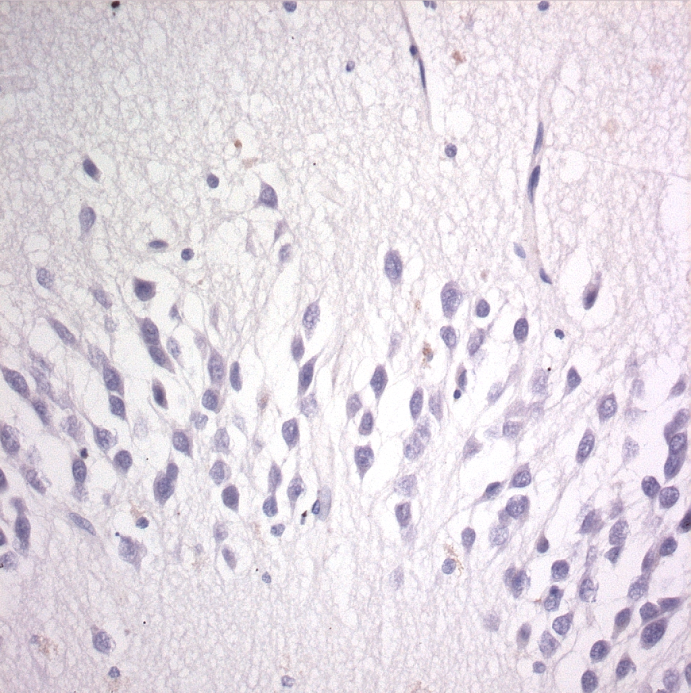


**50µm**

**3**

**2**

**1**

**0**

Supplement: Supplementary file 1 — Figure S1. Representative images of LAMP‐1 immunostaining in the dentate gyrus illustrating the grading system employed for scoring levels of immunostaining. [file NAN-45-244-s001.docx]

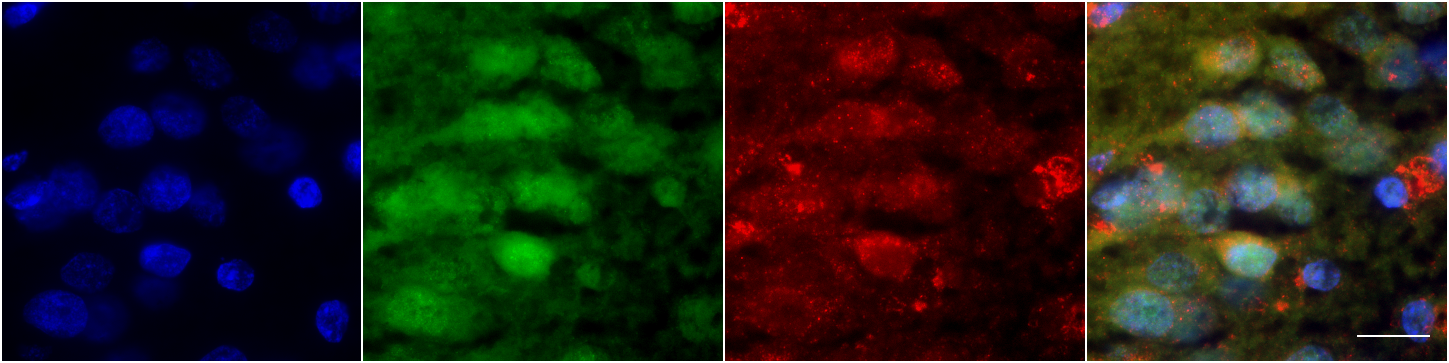

Supplement: Supplementary file 2 — Figure S2. Results of double immunofluorescence labelling of dentate gyrus granule cells in patient with FTLD‐TDP type B and expansion in C9orf72 (case #9). Reading from left to right, panels show DAPI labelling of nuclei (blue), TDP‐43 immunofluorescence (green), LAMP‐1 immunofluorescence (red) and merge of all the three images. Red granules representing ALP structures seen in LAMP‐1 panel are not identified in TDP‐43 panel where only nuclei are visible. In the merged image, ALP structures remain red showing no colocalization with TDP‐43, whereas nuclei are seen to be cyan in colour due to the merging of DAPI and TDP‐43 fluorescence. Scale bar indicates 10 μm. [file NAN-45-244-s002.tif]
